# A New Approach for the Modification of Paper Surface Properties Using Polyoxometalates

**DOI:** 10.3390/ma3010201

**Published:** 2010-01-07

**Authors:** Mikhail S. Saraiva, José A. F. Gamelas, António P. Mendes de Sousa, Bruno M. Reis, José L. Amaral, Paulo J. Ferreira

**Affiliations:** 1 Departamento de Engenharia Química, Universidade de Coimbra, Pólo II – R. Sílvio Lima, 3030-790 Coimbra, Portugal; E-Mail: mikhasa@gmail.com (M.S.S.); 2 RAIZ – Instituto de Investigação da Floresta e Papel, Quinta de São Francisco, Apartado 15, 3801-501 Eixo, Portugal; E-Mails: mendes.sousa@portucelsoporcel.com (A.P.M.d.S.); reisbruno24@gmail.com (B.M.R.); jose.luis.amaral@portucelsoporcel.com (J.L.A.)

**Keywords:** paper functionalization, polyoxometalates, cationic starch, surface sizing, coating, inkjet printing

## Abstract

A new approach for the chemical modification of the surface of paper based on the application of colloidal mixtures containing cationic starch and polyoxometalates on uncoated base paper is presented. Polyoxometalates with the Keggin-type structure and physical properties similar to those presented by coating pigments, namely H_3_PW_12_O_40_·23H_2_O, H_4_SiW_12_O_40_·24H_2_O, and K_7_PW_11_O_39_·9H_2_O, have been used in order to improve the quality of inkjet printing. The analysis of the different samples by FTIR-ATR spectroscopy showed the presence of the polyoxometalates (and the cationic starch) on the top surface of the paper. In addition, the determination of surface energy parameters, namely the polar component (σ_s_^p^) and the dispersive component (σ_s_^d^) of the surface energy, by contact angle measurements revealed that, for the new samples, the polar component level was much higher than that of the uncoated base paper. The quality of inkjet printing, evaluated by parameters such as the gamut area and the optical density, was considerably improved by these surface treatments.

## 1. Introduction

The treatment of the paper surface in order to control surface properties relevant to printing, such as surface energy, smoothness, porosity, and chemical properties, has been thoroughly explored. In this context, the modification of the paper surface properties should lead to a good paper-ink interaction (in inkjet or offset printing) in order to fix the ink colorants on the paper surface (without spreading) and to allow rapid absorption or evaporation of the ink vehicle. Therefore, a good affinity between the dyes (or pigments) used as colorants and the chemical compounds used in the paper surface treatment is desirable.

Two general approaches for the treatment of the paper surface are usually considered: surface sizing and coating. Surface sizing is usually performed by the application of an aqueous suspension of cationic starch alone or combined with a synthetic polymer, such as poly(styrene-co-acrylate) or poly(styrene-co-maleic anhydride), among others [1,2]. The coating processes use a different type of chemical formulation which includes a pigment (with kaolin, calcium carbonate, titanium dioxide, aluminum oxide, and silica being the most common ones used), a binder (mostly polyvinyl alcohol or latexes such as styrene butadiene), a co-binder (e.g., carboxymethylcellulose), a thickener (e.g., carboxymethyl cellulose) and a dispersant (a cationic polymer additive) [3,4,5,6,7,8,9,10,11]. Recently, sericite, a mineral composed mainly of silicon dioxide and aluminum oxide has also been proposed as a potential coating pigment [12].

Regarding paper functionalization, extensive work has also been carried out for the enhancement of paperboard surfaces used in food packaging, as recently reviewed by Andersson *et al.* [13]. For that purpose, several biopolymers [including starch and cellulose derivatives, chitosan, alginate, and poly(lactic acid)], as well as nanomaterials (montmorillonite) have been used to improve the water vapour and oxygen barrier properties, as well as water and grease resistance [13]. Besides the classical uses of paper, there is a large range of applications that can be envisaged for modified papers or new paper-based materials, depending on the type of chemical functionalization achieved at the paper surface. For instance, new catalytic or antibacterial functions may be designed if suitable substances, possessing specific properties, are efficiently immobilized at the paper surface, without losses of the strength and structural properties of the paper matrix.

**Figure 1:**
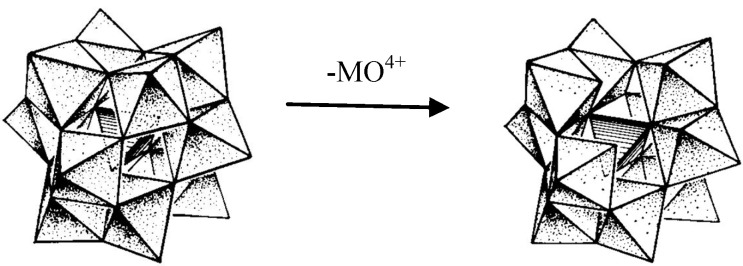
Polyhedral representation of the structure of the polyoxometalates α-[XM_12_O_40_]^p-^ and α-[XM_11_O_39_]^(p+4)-^ (X = P, Si; M = Mo, W).

In the present study, the use of polyoxometalates, never tested before for the modification of the paper surface, is addressed. Polyoxometalates (or polyoxoanions) are metal-cluster anions with the general formulae [M_x_O_y_]^m-^ or [X_z_M_x_O_y_]^n-^, where z ≤ x, and M stands for Mo, W, or V, and X is a variable element. They present unique structural and electronic properties which make them appealing in several fields, ranging from molecular architecture and catalysis to materials science [14,15,16,17,18,19,20]. In particular, polyoxometalates with the Keggin structure, α-[XM_12_O_40_]*^p^*^−^ (Figure 1), where X stands for P or Si, and M stands for Mo or W (Figure 1) are well known to form inorganic/organic hybrid associations and to strongly interact with protonated organic species derived from *N*-containing heterocycles, protonated aliphatic and aromatic amines, and with quaternary ammonium groups, among others [21,22,23,24,25]. This characteristic may be used to explore new ways of paper functionalization for inkjet printing. The removal of one MO^4+^ group from the Keggin anion gives rise to the corresponding monolacunary species, α-[XM_11_O_40_]^(p+4)−^ (Figure 1).

A new methodology consisting in the application of a colloidal suspension (with adequate rheology) containing cationic starch and a polyoxoanion at the paper surface, is presented. Polyoxometalate compounds (H_3_PW_12_O_40_·23H_2_O, H_4_SiW_12_O_40_·24H_2_O, and K_7_PW_11_O_39_·9H_2_O) with properties similar to those of coating pigments such as high reflectance in all the visible wavelength range, brightness, solubility (or good mixing) in water, compatibility with the other components of the formulation used in the surface treatment (only cationic starch in this study) were chosen with the objective of increasing the quality of inkjet printing.

## 2. Results and Discussion

### 2.1. Treatment of the Paper Surface with Cationic Starch and Polyoxometalates

**Figure 2:**
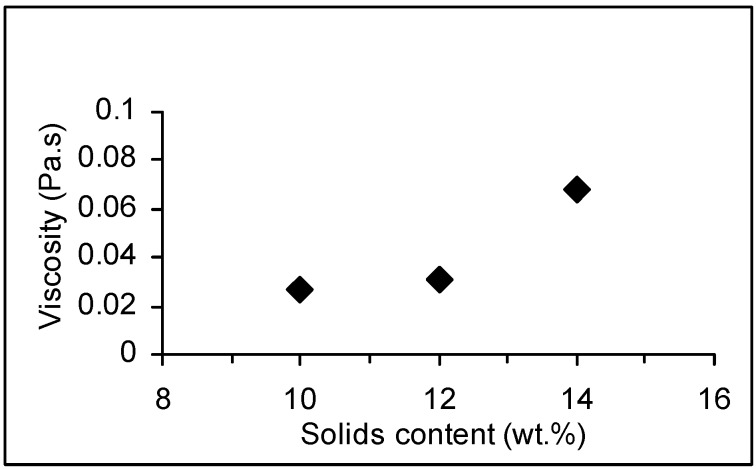
Viscosity at the maximum shear rate (600 s^-1^) of the cationic starch suspensions (50*°*C) *vs.* solids content.

A preliminary study of the viscosity of the cationic starch suspension was made at three different amounts of solids content (10%, 12% and 14 wt %). As shown in Figure 2, the hot suspensions (50 *°*C) of cationic starch with solids contents of 10 wt % and 12 wt % have not very different viscosities. At the solids content of 14 wt % a great increase of the viscosity to values near 70 mPa.s is observed. Given the reference value for some industrial applications (around 35 mPa.s), suspensions having 12 wt % of cationic starch were used in all subsequent experiments [26].

Due to the high solubility of H_3_PW_12_O_40_·23H_2_O and H_4_SiW_12_O_40_·24H_2_O in water, even at low temperature, and to their strong acid behaviour [17], there is a significant decrease of the pH from about 6 to 2–2.5 after the addition of those compounds to the cationic starch suspension (see also Experimental section 3.2 and Experimental section 3.3). The compound K_7_PW_11_O_39_·9H_2_O is also quite soluble in water, but, as expected, its addition to the starch suspension does not alter the pH of the suspension (near 6.0).

It should be noted that the cationic starch used to prepare the colloidal suspension has a very low content of epoxypropyltrimethylammonium groups ([C_3_H_6_ON(CH_3_)_3_]^+^) (based on the nitrogen analysis it was determined that the substitution degree of the quaternary ammonium groups in the starch structure is 4%, *i.e.,* per each 100 monomer units, 96 are glucose and the other four are substituted by the cationic groups). Considering the relative amounts of cationic starch and polyoxometalate solids used in each formulation for the treatment of the paper surface, 80/20 w/w, respectively (Experimental section 3.2 and Experimental section 3.3), the molar ratio between the epoxypropyltrimethyl-ammonium groups and the polyoxoanions is estimated to be between 3 and 3.5.

### 2.2. Characterization of the New Paper Samples by FTIR-ATR

**Figure 3:**
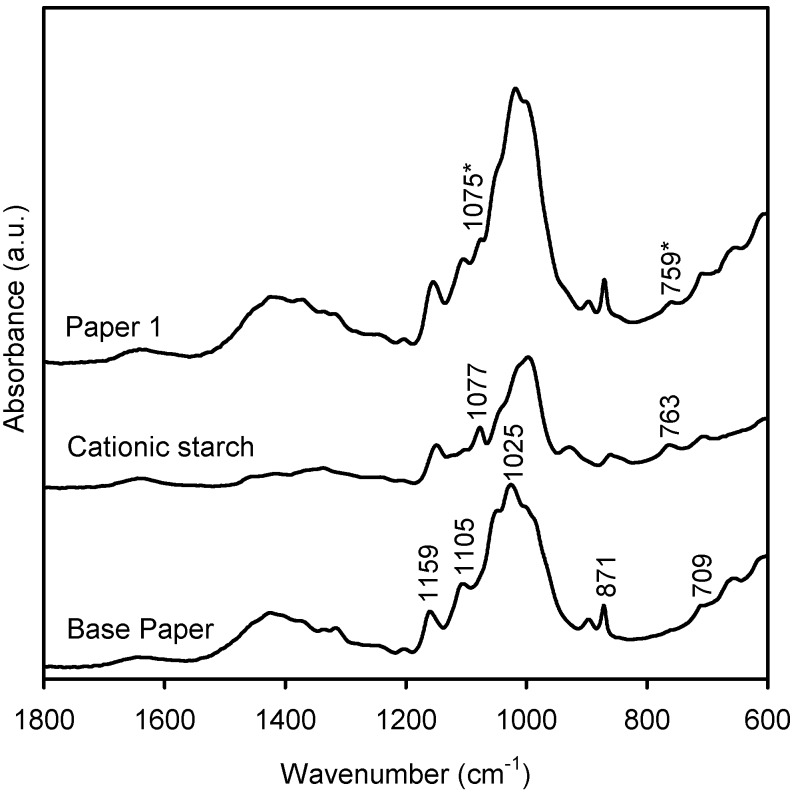
FTIR-ATR spectra of base paper, cationic starch, and paper 1 (treated with cationic starch).

**Figure 4:**
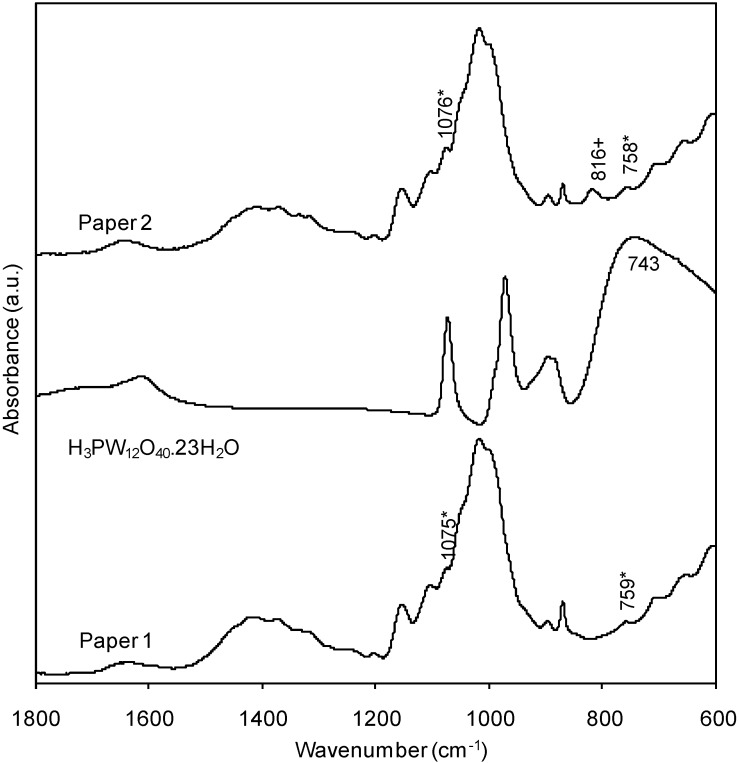
FTIR-ATR spectra of paper 1, H_3_PW_12_O_40_·23H_2_O, and paper 2 (treated with cationic starch and H_3_PW_12_O_40_·23H_2_O).

**Figure 5:**
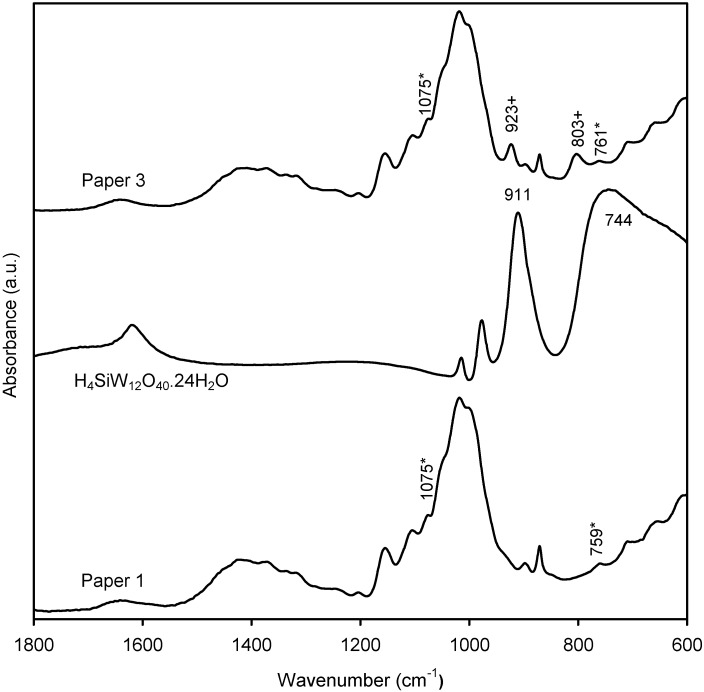
FTIR-ATR spectra of paper 1, H_4_SiW_12_O_40_·24H_2_O, and paper 3 (treated with cationic starch and H_4_SiW_12_O_40_·24H_2_O).

Infrared spectra of the surface of the base paper and of the four new paper samples (Experimental section 3.3), and of the cationic starch and polyoxometalates used in the treatment of the paper surface, were obtained. Some representative spectra are shown in Figure 3, Figure 4 and Figure 5. The infrared spectrum of the base paper (Figure 3) shows several bands due to calcium carbonate [27], cellulose, and hemicelluloses [28], as reported before [29]. The infrared spectrum of the paper treated with cationic starch (paper 1) shows two bands at 1,075 and 759 cm^-1^, not observed at the surface of the base paper, which are due to the presence of the cationic starch. These two bands are attributed to the C1-H bending and C-C stretching, respectively [30,31].

The FTIR-ATR spectrum of the surface of the paper obtained after treatment with the formulation containing H_3_PW_12_O_40_·23H_2_O and cationic starch is shown in Figure 4. The infrared spectrum of H_3_PW_12_O_40_·23H_2_O presents four main bands at 1,074, 972, 896 and 743 cm^-1^, corresponding to ν_as_(P-O), ν_as_(W-O), ν_as_(W-O_b_-W) and ν_as_(W-O_c_-W) (O_b_ and O_c_ denote oxygen atoms from octahedra sharing edges and corners), respectively [32]. For the paper treated with the mixture having 80/20 (w/w) of cationic starch and H_3_PW_12_O_40_·23H_2_O, respectively (paper 2) a new band, not observed at the surface of the paper treated only with cationic starch is observed at 816 cm^-1^. This band is attributed to ν_as_(W-O_c_-W), and is shifted to higher wavenumber by about 70 cm^-1^ in comparison to the analogous band observed in the spectrum of H_3_PW_12_O_40_·23H_2_O. Differences within this range have been observed in salts of Keggin anions with different counter-cations [32], as, for instance, when comparing the infrared absorption due to ν_as_(W-O_c_-W) in TBA_3_PW_12_O_40_ (800 cm^-1^) (TBA = tetra-*n*-butylammonium) with that of H_3_PW_12_O_40_·nH_2_O (around 740 cm^-1^). Certainly, the epoxypropylammonium groups, which are also quaternary ammonium groups (as TBA), the macromolecular structure of starch and the paper structure, are responsible for an analogous effect. The other polyoxometalate bands, in the 850–1,100 cm^-1^ range, are not observed at the surface of the treated paper because of their lower relative intensity, high absorbance of the paper matrix in this frequency region and also due to the low pick-up of the chemicals used in the paper surface treatment.

Concerning paper 3 (the one obtained by the treatment with H_4_SiW_12_O_40_·24H_2_O and cationic starch) the corresponding FTIR spectrum is presented in Figure 5. FTIR spectrum of H_4_SiW_12_O_40_·24H_2_O has four main bands at 1,015, 977, 911 and 744 cm^-1^ due to the ν_as_(Si-O), ν_as_(W-O), ν_as_(W-O_b_-W) and ν_as_(W-O_c_-W) vibration modes [32]. Two new bands not observed at the surface of the paper treated only with cationic starch appear at 923 and 803 cm^-1^, which are due to the ν_as_(W-O_b_-W) and ν_as_(W-O_c_-W) stretching vibrations, respectively. The band due to ν_as_(W-O_c_-W) is again shifted of about 60 cm^-1^ to a wavenumber higher than that observed in the spectrum of H_4_SiW_12_O_40_·24H_2_O.

The infrared spectrum of the paper 4 surface, obtained by treatment with the formulation containing cationic starch/K_7_PW_11_O_39_·9H_2_O, also reveals one new band not observed in the spectrum of the paper treated with cationic starch, at 810 cm^-1^, confirming the presence of the polyoxometalate on the paper surface.

### 2.3. Surface Energy Parameters

The contact angles of the five papers with water as well as the surface energy parameters computed by using the Owens and Wendt model [33] are presented in Table 1. The paper that exhibits the highest water contact angle is the base paper (102*°*) revealing a hydrophobic character. For all the paper samples treated with cationic starch and polyoxometalates, and for that treated only with cationic starch, the water contact angles are in the range of 30–50*°*, *i.e.,* are significantly lower. These results are not unexpected because the cationic starch molecules are polar (hydrophilic) and the polyoxoanions species typically have very low hydration energy [16]. The paper sample that presented the lowest water contact angle (30*°*) was that obtained by the treatment with the mixture containing 80/20 (w/w) of cationic starch and K_7_PW_11_O_39_·9H_2_O, respectively (paper 4). The slightly different water contact angles observed for the treated paper samples reflect the different chemical affinity of the paper surfaces to the water molecules which should depend mostly on the type and charge of the polyoxoanions used for the surface treatment, and on the counter-cations provided at the surface (H^+^, K^+^). For instance, when comparing the water contact angles of paper 2 and paper 3, treated with cationic starch/H_3_PW_12_O_40_ and cationic starch/H_4_SiW_12_O_40_, respectively, the water contact angle of paper 3 is lower since the [PW_12_O_40_]^3-^ and [SiW_12_O_40_]^4-^ anions have similar structure, but the latter anion has a higher charge density.

**Table 1:** Water contact angles and surface energy parameters determined for the paper samples.

Paper samples	Water contact angle (*°*)	Surface energy, σ_s_ (mN/m)	Polar component, σ_s_^p^ (mN/m)	Dispersive component, σ_s_^d^ (mN/m)	Correlation (R^2^)
Base paper	101.7 ± 2.2	47.4	0.1	47.3	0.660
1	38.2 ± 1.8	73.8	72.7	1.1	0.992
2	49.7 ± 2.3	59.6	56.9	2.7	0.991
3	42.2 ± 3.9	73.7	73.2	0.48	0.994
4	29.7 ± 2.4	87.4	87.2	0.21	0.988

In general, with the exception of the base paper, very good correlations were obtained for the determination of the surface energy parameters using the Owens and Wendt model [33] (Table 1). In the case of the base paper, the values determined are acceptable, since a close value for σ_s_^d^ was obtained by using the Fowkes model [34] (42.1 *vs.* 47.3 mN/m). The base paper is the one that presents the lowest polar component of the surface energy (around 0). For the paper samples obtained after the surface treatment, the σ_s_^p^ values are always significantly higher and the σ_s_^d^ values significantly lower in agreement with the smaller contact angles with water. Again, the differences between the σ_s_^p^ (and σ_s_^d^) values determined are mainly due to the different polyoxometalates and the corresponding counter-cations used in the treatment of the paper surface, as pointed out above when comparing the water contact angles. Accordingly, the paper having the highest surface energy and the highest polar component of the surface energy was paper 4.

### 2.4. Printability

**Figure 6:**
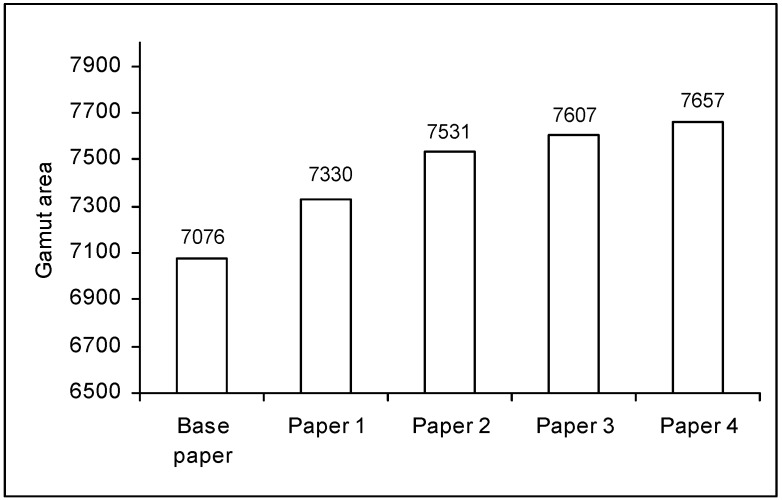
Gamut areas for the different paper samples.

The gamut area results obtained for the different paper samples are presented in Figure 6. As can be seen, the paper samples obtained by the surface treatment exhibit gamut areas significantly higher than that of the base paper, which is an indication of better colour reproducibility. Furthermore, the polyoxometalates have a positive impact in the gamut area, since the corresponding values obtained for the papers 2, 3 and 4 are higher than that of paper 1.

The values of the optical density (Table 2) are higher for the treated paper samples, with a few exceptions. For the black and magenta inks all the treated papers present slightly higher values in comparison with the base paper. As for the cyan and yellow, there is no clear tendency. However, it can be concluded that paper 2, 3 and 4 exhibit a better behaviour regarding the cyan, magenta and yellow (dyes) whereas paper 1 is the best regarding the black optical density.

**Table 2:** Optical density for distinct colours.

Paper samples	Cyan	Magenta	Yellow	Black 0
Base paper	0.935	0.91	0.968	1.376
1	0.897	0.922	0.958	1.482
2	0.951	0.924	1.029	1.459
3	0.976	0.948	1.005	1.425
4	0.914	0.939	0.980	1.462

The results presented in Table 3 reveal that the surface treatment has a clear positive influence in reducing both the print-through and intercolor bleed (the values for these parameters should be as low as possible, for better inkjet printing quality). A slight increase of the dot diameter, on the other hand, confirms a better retention of the inks at the surface. However, the role of the polyoxometalates is not evident. The values of the black line width may be considered similar to all samples.

**Table 3:** Print-through, line and dot printing quality parameters.

Paper samples	Print-through [0.0.0]	Line width, (black, μm)	Intercolor bleed	Dot diameter (black, μm)	Dot diameter (magenta, μm)
Base paper	1.31	474	50.1	507	409
1	0.53	465	37.4	527	419
2	0.70	480	39.4	529	413
3	0.42	519	43.3	551	426
4	0.64	480	36.4	528	416

According to all the inkjet printing quality parameters that were evaluated, paper sample 4 has a slightly better performance, considering its higher gamut area, higher optical density, lower intercolor bleed and low print-through. Paper samples 2 and 3 also have a good performance, being, for some parameters, better than paper 1, treated only with cationic starch. In general, the addition to the cationic starch of specific polyoxometalates for the paper surface treatment, improve printing quality.

In terms of gamut area, the values obtained in this study are slightly higher than those reported in the literature for paper samples surface sized with mixtures of cationic starch and different types of copolymers [2]. As for the optical density, the values are comparable to those reported for surface sizing [2], and for paper coating using silica or aluminum oxide pigments [8]. Recently, Nilsson and Fogden, reported gamut areas of 8,000–11,000 for a fine paper coated with formulations containing polystyrene plastic as pigment (with nominal particle size of 0.14 μm) and polyvinyl alcohol or carboxymethyl cellulose as binders [11]. However, it should be noted that the printing quality parameters depend not only on the compounds used for the surface treatment (and obviously on the physical-chemical properties of the base paper), but also on many other factors, such as the printing inks composition, printer model and printing mode. Thus, the comparison with data obtained in different experimental conditions from those used in this study should be done with due caution.

### 2.5. Relation between Printability and Chemical and Surface Properties of the New Paper Samples

**Figure 7:**
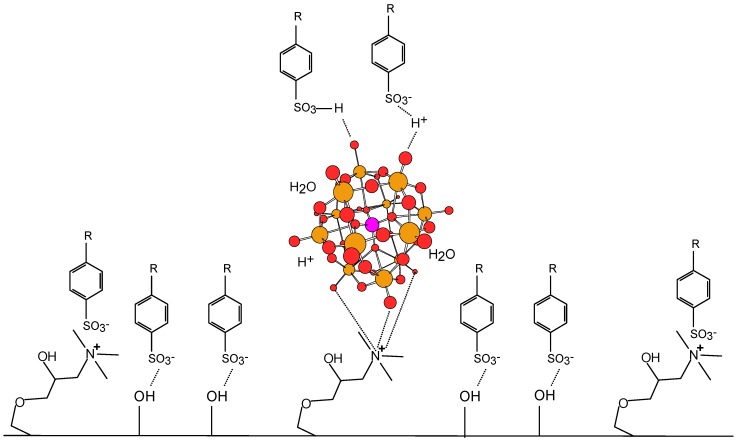
Schematic representation of the main chemical interactions which may occur at the paper surface functionalized with cationic starch (the cationic groups are the [C_3_H_6_ON(CH_3_)_3_]^+^) and heteropolyacids in the process of the ink dyes immobilization (the dyes are represented as an aromatic structure linked to a sulfonic group). Water molecules and protons around the polyoxoanion may be associated, forming other species [36].

Based on the alterations of the surface energy observed after the treatment of the base paper surface with cationic starch and polyoxometalates and on the presence of infrared bands at the paper surface due to both cationic starch and polyoxoanion species it is proposed that those two types of compounds are retained mostly at the paper top surface. Regarding cationic starch, this in agreement with recent studies by ToF-SIMS [35] and FTIR-ATR [29] for papers having high internal sizing degree. On the other hand, polyoxoanions of Keggin-type should be held at the paper surface, mainly by electrostatic bonding with the epoxypropyltrimethylammonium groups of substituted starch forming ionic pairs (Figure 7), as observed in related cases [23,25]. The slight excess in the number of the quaternary ammonium cations in comparison to that of polyoxoanions indicates that the major part of the cationic groups is not bound to the polyoxoanions (in each 100 glucose units about three of the four cationic groups present in the structure of cationic starch are not bound to the polyoxoanion).

The gamut area and the optical density increased for the treated papers relatively to the base paper, while the print-through and intercolor bleed decreased, all these trends showing a better printing quality. This may in part be attributed to the significantly higher polarity of the surface of the paper samples treated with cationic starch and polyoxometalate compounds. The highly polar surface provides a rapid diffusion of the ink liquid vehicle (mainly water and, in minor amounts, alcohols) into the “coating” layer, which in turn can contribute to the good immobilization of the ink dyes and pigments at the paper surface and their separation from the vehicle. Besides, despite the application of small amounts, the polyoxometalate play a relevant role and add to the positive influence of the cationic starch.

In inkjet, typically colorants are azo dyes having in their molecular structure sulfonic or carboxylic acid groups attached to an aromatic backbone. Other functional groups and substructures such as hydroxyl, amino, and nitrogen-containing heterocycles may be present as well [37,38]. Due to the high acidity of the sulfonic acid groups (and carboxylic acids in less extent), the corresponding dyes are predominantly anionic at the pH of the ink formulation (6.5–9). Therefore, the existence of cationic groups ([C_3_H_6_ON(CH_3_)_3_]^+^) at the paper surface, provide regions of high interaction with the sulfonic groups (anionic) of the inks. On the other hand, the polyoxoanions, located close to the quaternary ammonium groups of modified starch are not directly attracted to the anionic dyes structures. However, when the polyoxoanions are immobilized at the paper surface the counter-cations of the compounds used for the surface treatment are also available. For the heteropolyacid compounds used in the surface treatment of paper samples 2 and 3, there is also a concomitant release of protons to the medium (3 and 4 protons for the [PW_12_O_40_]^3-^ and [SiW_12_O_40_]^4-^ anions, respectively) due to their strong acidity (as shown above in 2.1). These protons should be close to the polyoxoanions structure, being attracted by the polyoxoanion and simultaneously attracting the sulfonic acid groups, the latter of which can be protonated (Figure 7). These interactions may contribute to the better ink dye retention at the paper surface in comparison to the use of cationic starch alone.

The results obtained with cationic starch and K_7_PW_11_O_39_·9H_2_O (paper 4) are pleasantly surprising since a high gamut area was achieved, although the optical density (namely of cyan and yellow) was not as high as that of papers 2 and 3. In this case, potassium cations together with the polyoxoanions are released to the paper surface. It is unlikely that potassium ions have the same affinity to the anionic dyes as the protons, although present in higher number (seven cations per one anion). However, paper sample 4 proved to have the most polar surface of all paper samples (the polar component was about 13% higher than that of paper 1, treated only with cationic starch) which can facilitate the retention of some colorants.

## 3. Experimental Section

### 3.1. Materials

A commercial calendered uncoated paper (80 g/m^2^) produced with a *Eucalyptus globulus* Kraft pulp was used as the base paper for the surface treatments. Cationic starch solid (with a nitrogen content of 0.37%) and α-amylase solution were supplied by the industry. Cationic starch suspensions having solids content of 10%, 12% and 14 wt % (and without polyoxometalate) were previously analysed using a Rheometer Rheostress (Haake), with the objective of selecting the one with the most suitable viscosity for further experiments. For the improvement of inkjet printing quality, the polyoxometalate compounds considered were H_3_PW_12_O_40_·23H_2_O, H_4_SiW_12_O_40_·24H_2_O, and K_7_PW_11_O_39_·9H_2_O. The former two were supplied by Sigma-Aldrich and the latter was prepared as described by Haraguchi *et al.* [39].

### 3.2 Preparation of the Cationic Starch/Polyoxometalate Mixtures for the Paper Surface Treatment

Water (at 60 *°*C, 37 mL) was added to 10 g of cationic starch. After heating the mixture to 65 *°*C with vigorous stirring, more 20 mL of hot water (60 *°*C) was added and the heating was extended to 70 *°*C. Then, 3.3 μL of α-amylase was added and the mixture was heated again up to about 80 *°*C and left at this temperature for 5 min. The starch enzymatic conversion was stopped by the addition of 1.7 mL of a ZnSO_4_ solution (30 g/L). The colloidal suspension obtained was heated up to 90–92 *°*C, and left stirring at this temperature during 15 min [26]. After, it was cooled to 50 *°*C, 2.5 g of solid polyoxometalate compound was added in small portions and the resultant suspension left stirring for about 10 min. The cationic starch content was finally adjusted to 12.0 ± 0.1 wt % by the addition of hot water. The suspension was maintained at approximately 50 °C before its application on the paper surface.

**Table 4:** General characteristics of the mixtures used for the treatment of the paper surface and paper samples obtained.

Paper samples	Mixtures used in the surface treatment		Average pick-up (g/m^2^)^a^
	Relative amounts (w/w) of solid compounds	pH (T~50 *°*C)	
1	100% Cationic starch	5.9	2.73
2	80% cationic starch/ 20% H_3_PW_12_O_40_	2.3	2.92
3	80% cationic starch/ 20% H_4_SiW_12_O_40_	2.1	2.51
4	80% cationic starch/ 20% K_7_PW_11_O_39_	6.0	2.88

^a^ The pick-up was obtained as the average of two replicates which did not differ more than 0.5 g/m^2^.

### 3.3. Treatment of the Paper Surface

The surface formulations were applied in both sides of the paper surface using a Mathis laboratory coating device, following the procedure reported elsewhere [29]. The surface-treated samples were no further calendered. Four treated paper samples were obtained (Table 4).

The pick-up of the surface treatment was obtained according to the ISO 536 standard. The values were in the range of 2.5–3 g/m^2^ (Table 4).

### 3.4. Evaluation of the Paper Surface Properties

The FTIR spectra were obtained by using a Mattson 7000-FTIR spectrometer equipped with an ATR cell from PIKE Technologies. The spectra were registered with a resolution of 4 cm^-1^, 256 scans and a signal gain of 10, in the range 300–4,000 cm^-1^. The crystal used in the ATR cell was diamond with a refractive index of 2.4. An incident radiation at 45*°* was applied.

The contact angles of the paper samples with four distinct liquids (water, formamide, ethylene glycol, and propylene glycol) were measured with a DataPhysics OCA20 using the sessile drop method, in order to determine the surface energy (σ_s_) of each paper sample, and the corresponding polar (σ_s_^p^) and dispersive components (σ_s_^d^) by the Owens and Wendt method [33].

Inkjet printing quality was evaluated by measuring the optical density (cyan, magenta, yellow and black), gamut area, print-through and some line and dot quality parameters in a specified mask printed with a HP Deskjet F370-target IJ12 (plain paper type and normal print quality) on the paper samples.

Both the optical density, gamut area, and print-through, whose definitions may be found elsewhere [2,40,41] were assessed by using the spectrophotometer AVAMOUSE. In addition, line width, dot diameter, and intercolor bleed (a measure of the mixture between two adjacent colours) were evaluated. The determination of these parameters was performed in the QEA portable digital microscope equipped with the personal image analysis system (PIAS).

## 4. Conclusions

In the present work a new methodology for the functionalization of paper surface, aiming a better printability, was developed. This methodology is based on the use of colloidal suspensions containing cationic starch and polyoxometalates for the treatment of the paper surface. Several new paper samples were obtained, and characterized by FTIR-ATR spectroscopy and contact angle measurements. The results of these studies showed that, for the new samples, the polyoxometalates (and cationic starch) are present on the paper surface and modify the surface energy parameters at a significant extent, in comparison to the base paper. In fact, after the surface treatments, the paper surface became much more polar, being the most polar surface that of the paper treated with the suspension containing cationic starch and the polyoxometalate K_7_PW_11_O_39_.

The new paper samples were evaluated with regards to inkjet printing quality, and very interesting results were obtained. In general, the gamut area and the optical density increased in comparison to the base paper, while the print-through and intercolor bleed decreased. This accounts for the significantly higher polarity of the surface of the new samples in comparison to the base paper. The highly polar surface of all treated papers (σ_s_^p^ > 50 mN/m; σ_s_^d^ < 3 mN/m) enables the rapid diffusion of the ink solvent, which may contribute to an easier separation of the ink colorants from the solvent helping on their immobilization at the paper surface.

Comparison was made between the paper samples treated with mixtures of cationic starch and polyoxometalates (H_3_PW_12_O_40_, H_4_SiW_12_O_40_ and K_7_PW_11_O_39_ for samples 2, 3 and 4, respectively) and the paper sample treated only with cationic starch (sample 1). The better results of gamut area and optical density of cyan, magenta and yellow for the paper samples 2, 3 and 4 when compared to those of paper 1 were interpreted in terms of the chemical interaction of the components of the dyes-based ink with the compounds used to modify the paper surface. It is proposed that the polyoxoanions and the corresponding counter-cations, by increasing of the electrostatic interactions with the anionic dyes (including, in the cases of paper samples 2 and 3, the protonation of the ionized sulfonic or carboxylic acid groups present in the structure of ink dyes) provide a better retention of the colorants at the paper surface. Work is in progress to better understand the processes occurring at the paper-ink interface for these new paper samples functionalized with cationic starch and polyoxometalates.

Overall, the use of polyoxometalate compounds together with cationic starch represents a novel approach for the treatment of paper surfaces. The formulation has two components: cationic starch and polyoxometalate, and the alteration of the surface properties, as demonstrated, is possible with the application of only small amounts of the chemicals at the paper surface, *i.e.,* with low pick-up. Finally, considering the compounds that were used, it is expected that the method presented in this study for the modification of the paper surface has not any negative impact in terms of paper recycling and environment when compared with the conventional methods of surface sizing and coating used at industry. Additional specific studies will have to be carried out to access the possibility of paper recycling without significant losses of the cationic starch and polyoxometalate compounds during the deinking process.
